# Molecular evolution of sex-biased genes in the *Drosophila ananassae *subgroup

**DOI:** 10.1186/1471-2148-9-291

**Published:** 2009-12-16

**Authors:** Sonja Grath, John F Baines, John Parsch

**Affiliations:** 1 Department of Biology, University of Munich (LMU), Munich, Germany

## Abstract

**Background:**

Genes with sex-biased expression often show rapid molecular evolution between species. Previous population genetic and comparative genomic studies of *Drosophila melanogaster *and *D. simulans *revealed that male-biased genes have especially high rates of adaptive evolution. To test if this is also the case for other lineages within the *melanogaster *group, we investigated gene expression in *D. ananassae*, a species that occurs in structured populations in tropical and subtropical regions. We used custom-made microarrays and published microarray data to characterize the sex-biased expression of 129 *D. ananassae *genes whose *D. melanogaster *orthologs had been classified previously as male-biased, female-biased, or unbiased in their expression and had been studied extensively at the population-genetic level. For 43 of these genes we surveyed DNA sequence polymorphism in a natural population of *D. ananassae *and determined divergence to the sister species *D. atripex *and *D. phaeopleura*.

**Results:**

Sex-biased expression is generally conserved between *D. melanogaster *and *D. ananassae*, with the majority of genes exhibiting the same bias in the two species. However, about one-third of the genes have either gained or lost sex-biased expression in one of the species and a small proportion of genes (~4%) have changed bias from one sex to the other. The male-biased genes of *D. ananassae *show evidence of positive selection acting at the protein level. However, the signal of adaptive protein evolution for male-biased genes is not as strong in *D. ananassae *as it is in *D. melanogaster *and is limited to genes with conserved male-biased expression in both species. Within *D. ananassae*, a significant signal of adaptive evolution is also detected for female-biased and unbiased genes.

**Conclusions:**

Our findings extend previous observations of widespread adaptive protein evolution to an independent *Drosophila *lineage, the *D. ananassae *subgroup. However, the rate of adaptive evolution is not greater for male-biased genes than for female-biased or unbiased genes, which suggests that there are differences in sex-biased gene evolution between the two lineages.

## Background

Sex-biased genes, *i.e*. those that differ in expression level between males and females, may be subject to differing selective constraints depending on the sex in which they are expressed or they may experience conflicting selective pressures in males and females (reviewed in [1]). Previous studies of *Drosophila melanogaster *have shown that male-biased genes, especially those expressed in reproductive tissues, consistently exhibit high levels of adaptive protein evolution [2,3]. Genome-wide comparisons of the ratio of the nonsynonymous substitution rate to the synonymous substitution rate (*d*_N_/*d*_S_) also indicate that male-biased genes are more functionally divergent between closely-related *Drosophila *species and are less likely to have identifiable orthologs between distantly-related species than genes with female-biased or unbiased expression [2,4]. A limitation of these studies is that they rely on gene expression data from *D. melanogaster *for sex-bias classifications and, thus, are not informative with respect to differences in sex-biased gene expression or sex-biased gene evolution between lineages.

In contrast to the studies that focused primarily on *D. melanogaster*, a recent SAGE (serial analysis of gene expression) study found no accelerated rate of protein evolution for male-biased genes in *D. pseudoobscura *[5]. This study measured the rate of evolution by the proportion of nonsynonymous substitutions (*d*_*N*_) between species and confirmed a higher rate of protein evolution in genes that had male-biased expression in both *D. melanogaster *and *D. pseudoobscura*, but found no evidence for an increased rate of evolution of genes that had male-biased expression only in *D. pseudoobscura*. The latter genes were only about half as divergent as the former and showed evolutionary rates similar to those of female-biased and unbiased genes. These results suggest that patterns of sex-biased gene evolution may have changed since the split of the *D. melanogaster *and *D. pseudoobscura *lineages. To further investigate this possibility, we analyzed sex-biased gene expression and DNA sequence polymorphism in *D. ananassae*, a species within the *melanogaster *group that serves as an outgroup to all species within the *melanogaster *subgroup, but is more closely related to *D. melanogaster *than *D. pseudoobscura *[6,7].

*D. ananassae *is distributed throughout the subtropical and tropical regions of the world. In contrast to *D. melanogaster*, *D. ananassae *is a species displaying significant population structure [8,9]. The demographic history has been investigated previously, with the ancestral range of the species being defined as a region of Southeast Asia that existed as a single landmass (Sundaland) during the late Pleistocene around 18,000 years ago [8-10]. Ancestral populations are expected to have colonized Asia and the South Pacific since the last glaciation and human migration to Oceania.

In this paper, we use species-specific microarrays to investigate sex-biased gene expression in *D. ananassae *for a set of genes previously investigated in *D. melanogaster*. We find that 60% of these genes show conserved sex-bias, while 40% differ in their sex-bias classification between the two species. Using multilocus statistical tests that compare ratios of polymorphism and divergence at synonymous and nonsynonymous sites, we detect a general signal of adaptive protein evolution in *D. ananassae*. However, this signal is not stronger for male-biased genes than for female-biased or unbiased genes, which is consistent with there being differences in sex-biased gene evolution between the *D. melanogaster *and *D. ananassae *lineages.

## Results

### Sex-biased gene expression in *D. ananassae*

To investigate sex-biased gene expression in *D. ananassae*, we designed a species-specific microarray of PCR-amplified exon sequences from 148 genes (Additional file 1) whose orthologs had been previously classified as male-biased, female-biased or unbiased in *D. melanogaster *(see Methods). This set of genes was of particular interest because the vast majority (136 genes) had been studied at the population-genetic level in *D. melanogaster *and estimates of the rate of adaptive evolution in the *melanogaster *subgroup were already available [2,3]. After quality control, we obtained sufficient hybridization signal to reliably classify 60 genes (22 male-biased, 13 female-biased, and 25 unbiased). Of the remaining genes, we were able to classify 69 (21 male-biased, 10 female-biased, and 38 unbiased) using the *D. ananassae *whole-genome microarray data of Zhang *et al*. [11]. In total, 129 of the 148 genes (87%) were classified, with 43 male-biased, 23 female-biased, and 63 unbiased (Additional file 2).

**Figure 1:**
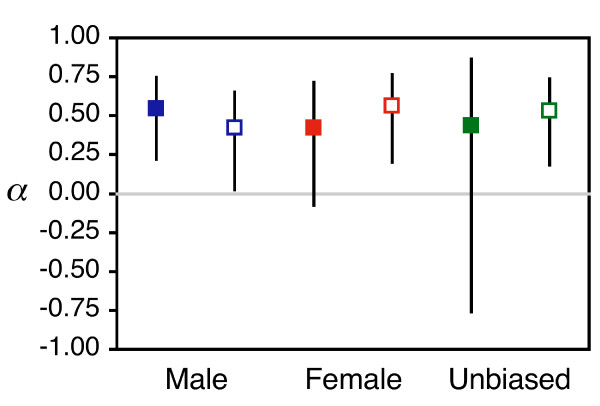
**Conservation of sex-biased gene expression between *D. melanogaster *and *D. ananassae***. Panel A shows the overall conservation of sex-biased expression between the two species. Panel B shows the conservation of the specific sex-bias classes, with the first letter indicating the bias in *D. ananassae *and the second letter indicating the bias in *D. melanogaster*. "M" indicates male-biased, "F" indicates female-biased, and "U" indicates unbiased expression. The area of the chart taken up by each category indicates the percentage of genes falling into that category. The number of genes in each category is given in parentheses.

To examine the conservation of sex-biased gene expression between species, we compared the above *D. ananassae *classifications to those previously determined for *D. melanogaster*. The majority of genes (59%) showed a conserved expression pattern, with the same bias in both species (Figure 1A). However, 37% of the genes were classified as sex-biased in only one species, and 4% showed the opposite sex-bias in the two species (Figure 1A). Overall, male-biased genes showed the greatest conservation: 72% of the genes with male-biased expression in *D. ananassae *also had male-biased expression in *D. melanogaster*, 65% of the genes with female-biased expression in *D. ananassae *also had female-biased expression in *D. melanogaster*, and 48% of the genes with unbiased expression in *D. ananassae *also had unbiased expression in *D. melanogaster*. Of the genes that were sex-biased in only one species, most (69%) were sex-biased in *D. melanogaster*, but unbiased in *D. ananassae *(Figure 1B). Three genes (*CG13690*, *CG3024*, *CG4593*) were male-biased in *D. ananassae*, but female-biased in *D. melanogaster*, while two genes (*CG12684*, *CG7387*) were female-biased in *D. ananassae*, but male-biased in *D. melanogaster *(Figure 1B). For *CG13690*, the molecular function is described as ribonuclease H activity and RNA binding. It is involved in the RNA metabolic process. The molecular function of *CG3024 *(*torp4a*) is described as ATP binding and unfolded protein binding. The gene is involved in protein folding. For *CG7387*, the function is given as unfolded protein binding and heat shock protein binding. It is also involved in protein folding. For the two remaining genes, no functions or biological processes are described. All described functions refer to what is known for *D. melanogaster*.

### Phylogenetic relationship of the focal species

**Figure 2:**
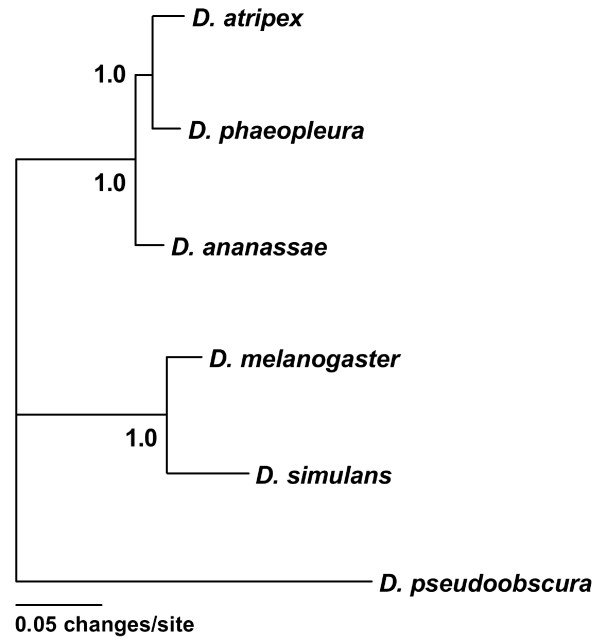
**Phylogeny**. 50% majority-rule consensus tree generated from concatenated amino acid sequences of 12 genes (3,446 sites) and Bayesian inference analysis (10,000 generations). Identical topologies were recovered in two independent runs of MrBayes [37]. Numbers at the nodes indicate posterior clade probabilities.

For analysis of evolutionary rates and tests for adaptive evolution, it is critical to have an appropriate outgroup species. Two recent molecular phylogenetic studies suggested that *D. atripex *and/or *D. phaeopleura *might serve as an appropriate outgroup to *D. ananassae *for these purposes [12,13]. However, these studies used a small number of loci that sometimes gave conflicting results. To further investigate the phylogenetic relationship of these species, we used the concatenated amino acid sequences of 12 genes that we sequenced in both *D. atripex *and *D. phaeopleura *(Additional file 3) and for which sequence data were available from *D. ananassae*, *D. melanogaster*, *D. simulans*, and *D. pseudoobscura*. The concatenated alignment of 3,446 amino acid positions was used to generate a phylogenetic tree by Bayesian inference (Figure 2). The tree topology was strongly supported (100% posterior clade probabilities for each node) and indicated that *D. atripex *and *D. phaeopleura *are more closely related to each other than either is to *D. ananassae*. Thus, both of these species can be used as an outgroup to *D. ananassae*. Furthermore, the divergence between *D ananassae *and *D. atripex*/*D. phaeopleura *is similar to the divergence between *D. melanogaster *and *D. simulans*, which facilitates the comparison of evolutionary patterns between the *melanogaster *and *ananassae *subgroups (see below).

### Levels of polymorphism and divergence

We surveyed DNA sequence polymorphism in a sample of 12 isofemale lines from Bangkok, Thailand for a subset of 43 genes used in our microarray analysis (Additional file 3). This set included 17 male-biased genes, 12 female-biased genes, and 14 unbiased genes. Fourteen of these genes differed in their sex-bias classification between *D. ananassae *and *D. melanogaster*. In addition, we sequenced all genes in a single strain of either *D. atripex *or *D. phaeopleura *to use as an outgroup. For 13 genes we were able to get sequences from both *D. atripex *and *D. phaeopleura*, revealing that both exhibit similar levels of divergence to *D. ananassae *(on average, 20% at synonymous sites and 2% at nonsynonymous sites) and that the choice of outgroup does not bias estimates of interspecific divergence (Table 1).

**Table 1:** Evolutionary rates of genes with male-, female-, and unbiased expression

Species	**Bias**^a^	Genes	***d***_*S*_	***d***_*N*_	***d***_*N*_**/*d***_*S*_
*Dmel *vs. *Dsim*	M	17	0.151	0.028	0.192
	F	16	0.128	0.019	0.154
	U	10	0.113	0.016	0.126
*Dana *vs. *Dat*	M	11	0.194	0.020	0.106
	F	8	0.222	0.017	0.071
	U	9	0.171	0.016	0.131
*Dana *vs. *Dph*	M	11	0.196	0.018	0.084
	F	8	0.160	0.019	0.118
	U	9	0.222	0.022	0.105

^a ^For the *Dmel *vs. *Dsim *comparison, sex-bias classifications come from *D. melanogaster *microarray data. For the other comparisons, sex-bias classifications come from *D. ananassae *microarray data. "M", male-biased; "F", female-biased; "U", unbiased.

Comparison of the divergence between *D. ananassae *and *D. atripex*/*D. phaeopleura *with the divergence between *D. melanogaster *and *D. simulans *for the different sex-bias classes of genes suggests that the accelerated rate of evolution of male-biased genes is limited to the *melanogaster *subgroup (Table 1). When comparing *D. melanogaster *and *D. simulans*, values of *d*_*N *_and *d*_N_/*d*_S _are consistently higher for male-biased genes than for female-biased or unbiased genes, which is not the case for comparisons of *D. ananassae *to either *D. atripex *or *D. phaeopleura *(Table 1).

However, there is no significant difference in *d*_*N*_/*d*_*S *_between male-biased genes on the different lineages (Mann-Whitney test, *P *> 0.05). It should be noted, however, that the number and identity of the genes are not constant among the three comparisons.

Levels of DNA sequence polymorphism in the Bangkok population of *D. ananassae *are comparable to levels in an ancestral African population of *D. melanogaster *(Table 2). On average, synonymous polymorphism is slightly higher in *D. ananassae *than in *D. melanogaster*, suggesting that the former has a slightly larger effective population size (*N*_e_). This is consistent with the Bangkok population representing an ancestral population of *D. ananassae *[8]. Taken together, levels of polymorphism and divergence suggest that our *D. ananassae *population and outgroup species are appropriate for comparison to the African *D. melanogaster *population with *D. simulans *as an outgroup.

**Table 2:** Intraspecific polymorphism in *D. melanogaster *and *D. ananassae*

Bias	Genes	*π*_*s*_^a^	*π*_*n*_^b^	π_*n*_/π_*s*_	*θ*_*s*_^c^	*θ*_*n*_^d^
	*D. melanogaster *(Zimbabwe, Africa)					
Male	17	0.0215	0.0015	0.0680	0.0234	0.0018
Female	16	0.0127	0.0017	0.1340	0.0135	0.0021
Unbiased	10	0.0149	0.0017	0.1115	0.0172	0.0020
All autosomal	29	0.0165	0.0013	0.0813	0.0175	0.0017
All X-linked	14	0.0176	0.0021	0.1198	0.0204	0.0025
All	43	0.0168	0.0016	0.0942	0.0185	0.0019
	*D. ananassae *(Bangkok, Thailand)					
Male	17	0.0197	0.0014	0.0723	0.0208	0.0017
Female	12	0.0234	0.0015	0.0628	0.0235	0.0018
Unbiased	14	0.0240	0.0017	0.0702	0.0249	0.0020
All autosomal	29	0.0236	0.0014	0.0590	0.0241	0.0017
All X-linked	14	0.0191	0.0018	0.0927	0.0203	0.0020
All	43	0.0221	0.0015	0.0687	0.0228	0.0018

^a ^The average number of nucleotide differences per synonymous site.

^b ^The average number of nucleotide differences per nonsynonymous site.

^c ^Mean nucleotide diversity (per site) at synonymous sites.

^d ^Mean nucleotide diversity (per site) at nonsynonymous sites.

We also investigated levels of synonymous polymorphism on the X chromosome relative to the autosomes in both *D. ananassae *and *D. melanogaster*. This provides an estimate of the effective population size of males and females in each species. The ratio is expected to be 3/4 if there is an equal number of breeding males and females in the population. If there is sexual selection acting on males, the male effective population size will be reduced and the X:autosome polymorphism ratio will be increased above 3/4. After standardizing polymorphism by divergence to correct for possible differences in mutation rate between the X and the autosomes, we find that the X:autosome polymorphism ratio is greater than 3/4 for both species (Table 3). To test the significance of this, we multiplied the X chromosomal values by 4/3 and compared them to the autosomal values with a Mann-Whitney test. For *D. melanogaster*, the difference was significant (*P *= 0.04), indicating that the original ratio was significantly greater than 3/4. For *D. ananassae*, an X:autosome polymorphism ratio of 3/4 could not be rejected (*P *= 0.13). There was no significant difference in the X:autosome polymorphism ratio between *D. ananassae *and *D. melanogaster *(*P *= 0.42).

**Table 3:** Synonymous polymorphism and divergence at X-linked and autosomal loci

	*D. ananassae*		*D. melanogaster*	
	X	Autosomal	X	Autosomal
*θ*_*s*_	0.0203	0.0233	0.0204	0.0175
*d*_*S*_	0.1860	0.1992	0.1418	0.1294
*θ*_*s*_/*d*_*S*_	0.1091	0.1210	0.1437	0.1352
X:A^a^		0.91		1.06

^a ^The ratio of X chromosomal to autosomal effective population size estimated from *θ*_*s*_/*d*_*S*_.

### McDonald-Kreitman tests

To evaluate the type of selection operating on individual genes, we applied single-locus McDonald-Kreitman (MK) tests [14] to the 43 genes for which we had polymorphism data from *D. ananassae *and divergence data from either *D. atripex *or *D. phaeopleura*. In total, six genes (14%) gave a significant MK test result (Additional file 3). Half of the significant genes departed from neutrality in the direction of positive selection (*i.e*. a relative excess of nonsynonymous divergence) (Table 4), while the other half departed from neutrality in a pattern consistent with either balancing or weak purifying selection (*i.e*. a relative excess of nonsynonymous polymorphism). These are likely to be cases of weak purifying selection, as average values of Tajima's *D *[15] are significantly negative at nonsynonymous sites (Table 5), which suggests that many segregating nonsynonymous polymorphisms are slightly deleterious.

**Table 4:** Genes with significant McDonald-Kreitman tests for positive selection

Gene	Bias^a^	*D*_*S*_^*b*^	*P*_*S*_^*c*^	*D*_*N*_^*d*^	*D*_*N*_^*e*^	*P*-value^f^	*D*_*S*_^*b*^	*P*_*S*_^*c*^	*D*_*N*_^*d*^	*P*_*N*_^*e*^	*P*-value^f^
		*D. ananassae*					*D. melanogaster*				
CG6980	MM	24	10	16	1	0.036	16	5	13	4	0.984
CG14717	UM	43	28	40	5	0.001	23	11	7	6	0.38
CG10750	UM	28	17	10	1	0.047	21	20	10	0	0.004
CG3085	MM	44	33	3	1	0.468	25	41	5	1	0.028
CG18341	UM	1	9	2	4	0.254	26	22	18	2	0.003
CG1314	MM	43	24	13	5	0.518	33	12	80	4	0.0004
CG9723	UU	64	10	16	7	0.076	36	18	61	5	0.0003

^a ^First letter indicates expression in *D. ananassae*, second letter expression in *D. melanogaster *(M = male-biased, F = female-biased, U = unbiased). The last three rows show all male-, female-, and unbiased genes according to the classification in *D. ananassae*.

^b ^The total number of synonymous fixed differences.

^c ^The total number of nonsynonymous fixed differences.

^d ^The total number of synonymous polymorphisms.

^e ^The total number of nonsynonymous polymorphisms.

^f ^*P*-value was determined by *G*-test when applicable, otherwise by Fisher's exact test.

**Table 5:** Average values of Tajima's *D*

Bias	Synonymous	Nonsynonymous
M	-0.21 (0.270)	-0.47 (0.046)
F	-0.08 (0.453)	-0.76 (0.040)
U	-0.19 (0.291)	-0.56 (0.030)

*P*-values (in parentheses) were determined as the proportion of 1000 simulations giving a Tajima's *D *value less than or equal to the observed.

A comparison of our MK test results to those from *D. melanogaster*/*D. simulans *[2,3] revealed that, in all but one case (*CG10750*), the individual genes showing significant evidence for positive selection (*i.e*. those with MK-test *P*-values less than 0.05) differed between the two lineages (Table 4). Most genes significant for positive selection were male-biased in at least one of the species. However, the proportion of positively-selected male-biased genes in *D. ananassae *(1/17 = 6%) was not as high as in previous studies of *D. melanogaster *where 7/33 (21%) of autosomal male-biased [3], and 7/17 (41%) of X-linked male-biased genes [2] gave significant MK tests for positive selection. Of the three genes with significant MK tests for positive selection in *D. ananassae*, one (*CG6980*) showed conserved male-biased expression between *D. ananassae *and *D. melanogaster*, while two (*CG14717 *and *CG10750*) were male-biased in *D. melanogaster*, but unbiased in *D. ananassae *(Table 4).

To further investigate expression class-dependent patterns of evolution, we constructed 12 groups of genes (Table 6). The first nine classes were partitioned with regard to sex-bias in *D. ananassae *and *D. melanogaster*. This allowed us to compare genes with conserved sex-bias between the two species with genes showing sex-bias in only one species or genes showing a reversal of sex-bias between species. The final three classes were partitioned solely on expression in *D. ananassae*, without regard to expression in *D. melanogaster*. Application of the MK test to the summed values of polymorphism and divergence within each group [16] revealed a significant departure from neutrality in the direction of positive selection for genes with conserved male-biased expression and for genes with male-biased expression private to *D. melanogaster*, whereas the female-biased and unbiased genes did not differ significantly from the neutral expectation (Table 6). The genes with male-biased gene expression private to *D. ananassae *also did not differ significantly from the neutral expectation.

**Table 6:** Summary of polymorphic and divergent sites

**Bias**^a^	Genes	***D***_*S*_	***D***_*N*_	***P***_*S*_	***P***_*N*_	*α*^a^	*P*_*S*_^b^	*P*_*N*_^b^	*α*^a, b^
MM	13	448	164	203	43	0.42**	122	19	0.57***
MU	2	44	6	5	6	-7.80**	4	2	-2.67
MF	2	77	39	14	8	-0.13	4	3	-0.48
FF	9	296	106	134	33	0.31	90	15	0.53**
FU	2	51	25	19	3	0.68	16	0	na(**)
FM	1	49	16	16	5	0.04	7	1	0.56
UU	6	163	34	97	19	0.06	60	4	0.68*
UM	3	72	52	54	10	0.74***	20	5	0.65*
UF	5	154	49	41	22	-0.69	37	10	0.15
M-	17	569	209	222	57	0.30*	130	24	0.50**
F-	12	396	147	162	41	0.32	123	16	0.65***
U-	14	389	135	192	51	0.23	117	19	0.53**

Symbols are the same as in Table 4.

^a^α = 1 - [(*D*_*S*_*P*_*N*_)/(*D*_*N*_*P*_*S*_)]. Asterisks indicate the significance of the summed data as determined by a *G*-test (*P < 0.05, **P < 0.01, ***P < 0.001).

^b ^Excluding low frequency (≤ 15%) polymorphisms.

### Estimation of the proportion of adaptive amino acid replacements

We also used a multi-locus version of the MK test to estimate *α*, the fraction of amino acid replacements between species that were fixed by positive selection [17]. For this, we divided the genes into six different groups. First, we considered male-, female-, and unbiased genes that had conserved sex-biased expression between *D. melanogaster *and *D. ananassae*. Second, we considered the genes according only to their bias in *D. ananassae*. We could not consider all 12 groups of genes mentioned above, because some of the groups contained too few genes to allow maximum likelihood analysis (*e.g*. there were only two genes with male-biased expression private to *D. ananassae*). Because the segregation of slightly deleterious nonsynonymous mutations can lead to a downward bias in the estimate of *α*, we excluded all polymorphisms (both synonymous and nonsynonymous) segregating at frequency ≤ 15% from our analysis [18].

**Figure 3:**
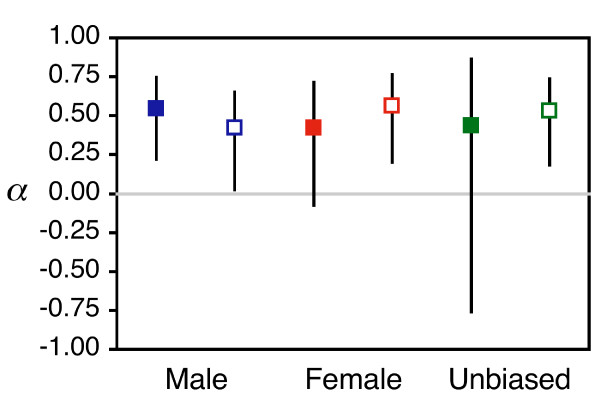
**Maximum likelihood estimates of the fraction of positively selected amino acid replacements (α)**. Values of *α *for genes with male-, female-, and unbiased expression were calculated using a maximum likelihood method [17]. Genes with conserved bias between *D. ananassae *and *D. melanogaster *are indicated with solid boxes, genes with bias according to classification in *D. ananassae *(*i.e*. including conserved genes and genes with bias private to *D. ananassae*) are indicated with open boxes. Low frequency polymorphisms (≤ 15%) were excluded. Error bars indicate 95% CI. Asterisks indicate genes with a significant signal of positive selection (*P < 0.05, **P < 0.01).

For the genes with conserved sex-biased expression between species, we observed a significant signal of positive selection only for those with conserved male-biased expression, although in all cases the mean estimate of a was greater than zero (Figure 3). However, when we considered the genes only by their sex-bias classification in *D. ananassae *we found evidence for adaptive evolution in all three classes of genes, with higher α estimates and more significant departures from neutrality detected for female-biased and unbiased genes than for male-biased genes (Figure 3). Thus, the increased rate of adaptive evolution seen for male-biased genes appears to be limited to those with conserved male-biased expression between the two species. For *D. ananassae*, estimates of *α *were 43%, 60%, and 53% for male-biased, female-biased, and unbiased genes, respectively.

## Discussion

Although sex-biased expression was conserved between *D. ananassae *and *D. melanogaster *for the majority of the genes we analyzed, a large minority (40%) showed a gain, loss, or reversal of sex-biased expression between species (Figure 1). In a previous microarray study, Ranz *et al*. [19] found that 20% of genes showed a gain, loss, or reversal of sex-biased expression between *D. melanogaster *and *D. simulans*. The higher percentage observed in the present study is likely attributable to the greater evolutionary distance separating *D. melanogaster *and *D. ananassae *(Figure 2), which provides more opportunity for expression changes. Consistent with this, a previous SAGE study found that 34% of genes changed their sex-biased expression pattern between *D. melanogaster *and the more distantly-related *D. pseudoobscura *[5].

Several aspects of our experimental design might also contribute to the gene expression patterns described above. First, the genes on our array were not a random set, but instead were enriched for those showing strong sex-biased expression in multiple, independent experiments in *D. melanogaster*. Thus, we might expect to see an overrepresentation of genes that are sex-biased only in *D. melanogaster*, and an underrepresentation of genes that are sex-biased only in *D. ananassae*. Indeed, this is what we find: of the 48 genes that are sex-biased in only one species, 33 (69%) are sex-biased in *D. melanogaster*, while only 15 (31%) are sex-biased in *D. ananassae *(Figure 1B). A second factor that may influence our results is that our *D. ananassae *classifications come from a combination of our own experimental data and those from a published whole-genome microarray study [11] and the two studies differ in their design and replication. Our own microarray experiments examined only a small set of genes, which allowed for many replicate probes per gene to be present on each array. We also enforced strict quality control to exclude genes with weak hybridization signal (which would otherwise be classified as unbiased). The use of the published whole-genome arrays allowed us to classify more genes, but the classification may be less reliable due to the lower replication and the increased problem of multiple testing that weakens the statistical analysis. Indeed, the whole-genome data classify a higher fraction of unbiased genes (68% *vs*. 33% for our custom arrays), which would be expected since the null hypothesis of these comparisons is that there is no difference in expression level between males and females. Also, it should be noted that the whole-genome arrays of Zhang *et al*. [11] only identified ~12% of the *D. ananassae *transcriptome as sex-biased, while the previous studies that our *D. melanogaster *classifications are based on identified 20-70% of the transcriptome as sex-biased [19-22]. This difference is most likely the result of differences in experimental design and statistical power between the studies, not an underlying difference in the amount of sex-biased expression between the two species, as Zhang *et al*. [11] also reported similarly low percentages of sex-biased genes in six other *Drosophila *species, including *D. melanogaster*.

Despite the presumably weaker power to detect sex-biased expression in *D. ananassae*, we observed 15 genes that were sex-biased in *D. ananassae*, but unbiased in *D. melanogaster *(Figure 1B). For 12 of these genes, the ancestral expression state could be inferred using microarray data from the outgroup species *D. pseudoobscura *[11] (Additional file 4). Interestingly, 11 of these 12 genes showed a match between the *D. melanogaster *and the *D. pseudoobscura *classification, suggesting that sex-biased expression was gained on the *D. ananassae *lineage in the vast majority of cases. The ancestral expression state could also be inferred for four of the five genes that showed a reversal of sex-bias between *D. melanogaster *and *D. ananassae*. Three of these genes were female-biased in *D. melanogaster *and *D. pseudoobscura*, but male-biased in *D. ananassae*. One gene was male-biased in *D. pseudoobscura *and *D. melanogaster*, but female-biased in *D. ananassae*. However, this gene (*CG7387*) also differed in its sex-bias classification between our custom microarrays (female-biased) and the whole-genome *D. ananassae *microarrays (male-biased) [11]. Thus, it is possible that the sex-biased expression of this gene is strain- or condition-dependent. This was the only such conflict between the two *D. ananassae *expression datasets, although there were 26 cases where a gene was classified as sex-biased in one dataset and unbiased in the other.

Our survey of DNA sequence polymorphism in *D. ananassae *is the largest performed to date in terms of number of loci investigated and the first to examine genes with sex-biased expression. Overall, the level of polymorphism in the *D. ananassae *population from Bangkok, Thailand is similar to that in an ancestral African *D. melanogaster *population, which is consistent with Bangkok being within the ancestral species range of *D. ananassae *[8]. When considering all loci we find that *D. ananassae *has slightly more synonymous polymorphism than *D. melanogaster *(Table 2), which suggest that the former has a larger *N*_e_. Consistent with this interpretation, nonsynonymous polymorphism and the ratio of nonsynonymous to synonymous polymorphism are lower in *D. ananassae *(Table 2). This is expected if most segregating nonsynonymous mutations are slightly deleterious, as is suggested by their negative values of Tajima's *D *(Table 4), because purifying selection is more effective at removing deleterious mutations when *N*_e _is large.

Multi-locus analyses of polymorphism and divergence indicate that adaptive protein evolution is prevalent in *D. ananassae*, with estimates of *α *in the range of 50-60% (Table 3 and Figure 3). These *α *values are remarkably similar to those estimated for other *Drosophila *species (reviewed in [23]), which suggests that there is a consistently high rate of adaptive protein evolution throughout the genus. However, despite the strong overall signal of positive selection acting on *D. ananassae *proteins, we do not see clear differences in the prevalence of adaptive protein evolution among male-, female-, and unbiased genes. This contrasts with previous results from *D. melanogaster *that indicated an increased rate of adaptive evolution in male-biased genes [2,3], but is consistent with the results of Metta *et al*. [5], who found accelerated rates of evolution (as measured by *d*_*N*_) for *D. melanogaster *male-biased genes, but not *D. pseudoobscura *male-biased genes. We see the same general trend for *d*_*N *_(and *d*_*N*_/*d*_*S*_) values in our data, which are highest for the male-biased genes of *D. melanogaster*, but lower for the male-biased genes of *D. ananassae *(Table 1). Taken together, these results suggest that male-biased genes have experienced an increase in the rate of adaptive protein evolution since the divergence of the *ananassae *and *melanogaster *subgroups (Figure 2).

The above results could be explained if sexual selection drives the evolution of many male-biased genes and the prevalence of sexual selection differs between the two subgroups. In ancestral *D. melanogaster *populations, X-linked synonymous polymorphism is significantly greater than 3/4 of autosomal synonymous polymorphism [24], which is expected if sexual selection acts on males (reviewed in [25]). We observe a similar pattern for *D. ananassae *(Table 3), suggesting that sexual selection does not differ greatly between the species. However, due to the relatively small number of *D. ananassae *loci, we cannot detect a significant difference between the observed X:autosome diversity ratio and the expected ratio of 3/4, nor can we detect a significant difference in this ratio between *D. ananassae *and *D. melanogaster*. Thus, we cannot exclude the possibility that sexual selection is weaker in the *D. ananassae *lineage. Mate-choice experiments, however, indicate that there is significant female mate preference in *D. ananassae *[26] and that male courtship song plays a major role in female mate discrimination [27]. Furthermore, it has been shown that *D. ananassae *males are subject to stronger intrasexual selection than females and that male mating success is correlated with morphological traits, such as body size and sternopleural bristle number [28]. These findings suggest that sexual selection plays an important role in the species' evolution.

The observed differences in male-biased gene evolution between *D. ananassae *and *D. melanogaster *could also be influenced by the particular genes that were investigated. Our initial gene set was enriched for genes that showed strong and consistent sex-biased expression across multiple *D. melanogaster *microarray experiments. On average, the subset of male-biased genes showed a 12-fold male-bias in *D. melanogaster*, but only a 5-fold male bias in *D. ananassae*. While it is difficult to directly compare male/female expression ratios across different experiments and microarray platforms, it is likely that most of the genes investigated had a stronger male-bias in *D. melanogaster *than in *D. ananassae*. If the degree of male bias is correlated with the rate of adaptive evolution across the genus, as it appears to be in the *melanogaster *subgroup [2], it could explain differences in male-biased gene evolution between the lineages. This possibility could be addressed in future studies focusing on genes with exceptionally strong male-biased expression in *D. ananassae*. However, there is not a significant correlation between *d*_*N*_*/d*_*S *_and the male/female expression ratio within our *D. ananassae *dataset (Pearson's *R *= 0.10, *P *= 0.52). In general, the female-biased genes included in our study showed weaker sex-biased expression than the male-biased genes in *D. melanogaster *(4-fold vs. 12-fold). This might explain why expression conservation between *D. melanogaster *and *D. ananassae *was greater for male-biased genes than female-biased genes (Figure 1), as highly biased genes are more likely to de detected as significant, while weakly biased genes are more likely to be non-significant and classified as unbiased.

## Conclusions

Although sex-biased gene expression is abundant in *Drosophila *species, the sex-biased expression pattern of many genes differs between species. Species-specific microarray data indicate that 26% of genes with a strong sex bias in *D. melanogaster *show no detectable sex bias in *D. ananassae*, while 12% of genes with unbiased expression in *D. melanogaster *show significantly sex-biased expression in *D. ananassae*. The accelerated rate of adaptive evolution seen for male-biased genes in *D. melanogaster *is not observed for male-biased genes in *D. ananassae*, which suggests that there are differences in sex-biased gene evolution between the two lineages. This is in agreement with a previous study on rates of protein evolution in *D. pseudoobscura *[5] and suggests that the rapid adaptive evolution of male-biased genes is unique to the *melanogaster *subgroup and not a general pattern in *Drosophilids*. Despite these differences, the overall signal of adaptive protein evolution is strong in *D. ananassae *(*α *≈ 50%) and is consistent with previous estimates throughout the genus.

## Methods

### Microarray analysis

To analyze sex-biased gene expression in *D. ananassae*, we designed a species-specific PCR-amplicon microarray. We began with a set of 148 genes that had previously been studied in *D. melanogaster *[2,3] and used the available *D. ananassae *genome sequence (Assembly August 2005; http://genome.ucsc.edu/[29]) to identify their orthologs and design PCR primers that amplify exonic sequences (mean length = 458 bp). A complete list of genes and PCR primers is provided in (Additional file 1). PCR products were purified using genPURE PCR 96 well filter plates (Genetix) and spotted on UltraGAPS slides (Corning) using a GeneMachines OmniGrid Accent microarrayer. *D. ananassae *genomic DNA (gDNA) was also spotted as a control. Eight replicates of each gene probe were spotted per array, with each replicate in a different subarray. 12-14 control gDNA probes were spotted per subarray.

For hybridization, we extracted total RNA from four- to five-day old males and females using Trizol reagent (Invitrogen) and the manufacturer's protocol. Two inbred strains of *D. ananassae *from Kota Kinabalu, Borneo were used for RNA extraction [8]. Reverse transcription was performed using 25 μg of total RNA and an anchored oligo(dT) primer. cDNA was labelled with fluorescent dyes (Alexa Fluor 555 and 647) using the amino-allyl labelling system (Invitrogen). Labelled male and female cDNA was competitively hybridized to the arrays for 20 hours at 42 C. Arrays were scanned using a Genetix aQuire 2-laser microarray scanner and the Genetix Qscan software. In total, we performed 12 replicate hybridizations. Six of these were biological replicates, *i.e*. from different RNA extractions performed at different times, while the other six were "dye-swap" replicates where we exchanged the fluorescent dyes used to label male and female cDNA within each biological replicate.

Prior to statistical analysis, normalization of the two dye channels was performed. Since our arrays contained only a subset of the genome with a non-random distribution of sex-biased genes, we used the gDNA spots as controls for normalization. The signal for each spot was calculated by subtracting the median local background from the mean spot intensity. If this result was negative for a given channel, that channel was assigned a value of 0.5 according to the MINIMUM approach of LIMMA [30]. For each replicate subarray, the red/green ratio was calculated for each of the gDNA control spots and the mean of these ratios was taken as the normalization factor. The raw red value of each spot was then multiplied by this factor to obtain the normalized red value used to calculate the corrected red/green ratio. Note that this is a local normalization, with a different normalization factor for each replicate subarray. As a quality control measure, only spots with a mean intensity at least 20% above the mean local background in at least one of the channels were used. For any one array, we required that at least half of the replicate spots per gene (in this case four) displayed adequate signal as defined above. The median red/green ratio (after normalization) of these replicate spots was used for statistical analysis using the BAGEL software [31,32]. To determine the *P-*value cut-off used to define significant sex-biased genes, we performed randomizations of the BAGEL input file to estimate the false-discovery rate (FDR) for the dataset. For classification of sex-biased genes, we used a *P*-value cut-off of 0.01, which corresponds to a FDR of 10%. All new microarray data have been deposited in NCBI's Gene Expression Omnibus [33] and are accessible through GEO Series accession number GSE19096 http://www.ncbi.nlm.nih.gov/geo/query/acc.cgi?acc=GSE19096.

### Fly strains, polymerase chain reaction and DNA sequencing

For the polymorphism survey, we used 12 inbred strains of *D. ananassae *from Bangkok, Thailand. Like the Kota Kinabalu strains used for the microarray experiments, these strains were included in a previous population genetic survey of intronic loci and found to be in the ancestral range of *D. ananassae *[8]. Thus, we expect little differentiation between these populations. We chose the Bangkok population for the polymorphism survey because a larger number of strains were available.

One strain each of *D. atripex *and *D. phaeopleura *(kindly provided by M. Schug) were used as outgroups. We used the *D. ananassae *genome (Assembly August 2005; http://genome.ucsc.edu/[34]) to design PCR primers flanking the coding sequence of 43 target genes. These genes were chosen to represent different categories of sex-biased gene expression on the basis of the microarray data described above. Following PCR, the amplified products were purified with ExoSAP-IT (USB, Cleveland, OH) and sequenced on both strands using BigDye version 1.1 chemistry and a 3730 automated sequencer (Applied Biosystems, Foster City, CA). The PCR primers were also used as sequencing primers in the polymorphism survey. A complete list of primers is provided in Additional file 5. For some genes, we were unable to get successful PCR or DNA sequence from all 12 *D. ananassae *strains (Additional file 3). The average number of strains sequenced per gene was 11 and we required at least eight sequences for a gene to be included in our analysis. All new DNA sequences have been submitted to the GenBank/EMBL databases under accession numbers FN546265-FN546780.

### Analysis of polymorphism and divergence

Sequences were edited using DNAstar (Madison, WI) and multiple alignments were calculated using MUSCLE [35]http://www.ebi.ac.uk/Tools/muscle/. Polymorphism and divergence statistics were calculated using DnaSP 4.5 [36]. For McDonald-Kreitman (MK) table data we used the number of segregating mutations instead of the number of segregating sites as some genes had sites with three segregating variants. For divergence, we considered only sites with fixed differences between all *D. ananassae *lines and a single *D. atripex *(or *D. phaeopleura*) sequence. Multilocus Tajima's *D *tests were performed using the HKA program http://lifesci.rutgers.edu/~heylab/heylabsoftware.htm. To calculate the fraction of positively selected amino acid substitutions, a, with the method of [17], we used the DoFE program http://www.lifesci.sussex.ac.uk/home/Adam_Eyre-Walker/Website/Software.html. For MK tests and a calculations, the *D. atripex *sequence was used as the outgroup whenever available. When the *D. atripex *sequence was not available, *D. phaeopleura *was used as the outgroup.

### Phylogenetic analysis

For 13 genes we were able to get sequences from both *D. atripex *and *D. phaeopleura *[Additional file 3]. In addition, we downloaded the amino acid sequences of *D. melanogaster*, *D. simulans*, *D. ananassae*, and *D. pseudoobscura *for the entire set of genes analyzed from Flybase http://flybase.org/. For one gene (*CG18418*), we were unable to get the orthologous sequence from *D. pseudoobscura*. This left us with 12 genes that could be aligned across all species (*CG2577, CG3004, CG3024, CG4593, CG6459, CG7840, CG8277, CG9135, CG10853, CG11379, CG15336*, and *CG15717*). Amino acid sequences of these 12 genes were concatenated for phylogenetic reconstruction Bayesian inference (BI), as implemented in MrBayes version 3.1.1 [37], was used to reconstruct the phylogeny with *D. pseudoobscura *set as the outgroup. For the BI analysis, two distinct 10,000 generation runs were conducted (three incrementally heated chains with model jumping between fixed-rate amino acid models were used and trees were saved to a file every 10 generations). Identical topologies were recovered from both runs. A burn-in period of 2,500 generations was determined graphically by plotting likelihood values for each sample. The results were presented in the form of a 50% majority-rule consensus tree in which trees corresponding to the burn-in period were discarded. Support for the nodes was given by posterior probability estimates of clades. Using model jumping, only the Jones (JTT) model [38] contributed to the final result with 100% posterior probability. This was also supported by the program ProtTest version 2.0 [39], which determines the best-fit substitution model for amino acid data under a likelihood framework. Within this framework, the Akaike information criterion [40] selected the JTT [38] model including the proportion of invariable sites and the gamma distribution of rate variation among sites (JTT + I + G) as the best-fitting model with an Akaike weight of 0.54. The second-best model was JTT+G with an Akaike weight of 0.46. Trees were visualized with TreeView [41].

## Authors' contributions

SG carried out the experiments, analyzed the data, and drafted the manuscript. JFB participated in the design of the study and helped with sequence analysis and microarray preparation. JP conceived of the study, participated in its design and coordination, and performed statistical analysis of microarray data. All authors read and approved the final manuscript.

## Additional material

All additional material is provided on a small webpage that can be downloaded as a zip-archive. All files can be accessed through *index.html*, which can be viewed with any standard internet browser (Additional File 6)

## Supplementary Material

Additional file 1 **Primers for PCR-amplicon microarrays**. Primers were designed to amplify single exons of protein-coding genes using the *D. ananassae *genome assembly from August 2005 http://genome.ucsc.edu/. Given is the length of the amplified fragment. The dataset contains 148 candidate genes plus two highly-expressed genes (*Adh*, *RpL23*) that were used as controls. Click here for file

Additional file 2 **Sex-biased gene expression in *D. ananassae *compared to *D. melanogaster *and *D. pseudoobscura***. The degree of sex-biased gene expression is given as the ratio of male/female expression. For *D. ananassae*, gene expression ratio was determined from up to two independent experiments. For *D. melanogaster*, expression data from up to three independent experiments were used. Click here for file

Additional file 3 **Comparison *D. ananassae*/*D. melanogaster***. For 43 genes, intraspecific polymorphism data were available for both *D. melanogaster *(Zimbabwe, Africa) and *D. ananassae *(Bangkok, Thailand). For *D. melanogaster*, divergence was determined to *D. simulans*. For *D. ananassae*, *D. atripex *and/or *D. phaeopleura *were used for calculating divergence. Click here for file

Additional file 4 **Inference of ancestral sex-biased expression state of genes differing in expression between *D. melanogaster *(*Dmel*) and *D. ananassae *(*Dana*), using *D. pseudoobscura *(*Dpse*) as the outgroup**. For 47 genes showing conflicting sex-biased classifications between *D. melanogaster *and *D. ananassae*, we inferred the ancestral expression state using microarray data from *D. pseudoobscura*. Click here for file

Additional file 5 **PCR and sequencing primers**. Primers were designed to amplify genomic regions of 43 protein-coding genes. The PCR primers were also used as sequencing primers, with internal primers designed when necessary. Click here for file

Additional file 6 **All additional material**. All files can be accessed through *index.html*, which can be viewed with any standard internet browser. Click here for file
