# Supplementary material for: Molecular evolution of sex-biased genes in the Drosophila ananassae subgroup
Source: BMC Evol Biol. 2009 Dec 16;9:291. doi: 10.1186/1471-2148-9-291 (PMC2809073; doi:10.1186/1471-2148-9-291)
Supplement: Additional file 1 — Primers for PCR-amplicon microarrays. Primers were designed to amplify single exons of protein-coding genes using the D. ananassae genome assembly from August 2005 http://genome.ucsc.edu/. Given is the length of the amplified fragment. The dataset contains 148 candidate genes plus two highly-expressed genes (Adh, RpL23) that were used as controls. [file 1471-2148-9-291-S1.pdf]

Additional file 1 – Primers for PCR-amplicon microarrays

Gene	Chro.[a]	Exp.[b]	Forward primer (5' - 3')	Reverse primer (5' - 3')	Length[c]
CG3481	2L	Control (*Adh*)	gagtgataatgtccgacg	gaacttacggctagggagc	653
CG3661	2R	Control (*RpL23*)	ccctcttctacccacaggtc	gcctagagaagatccacc	334
CG2867	3R	Female	acttgccttcgctggtggc	gatcaagccgcggtcacactgg	473
CG9383	3L	Female	tcggttgcaaatctgatccg	ccagaccattactgttaccg	527
CG9273	2L	Female	tgcatgccaaacatctcg	ccaacaggccacattaatgc	376
CG4973	3R	Female	gtatgtggcaatttaccgcc	ccactcacttggttaccac	784
CG1239	3R	Female	acggcgtaaaaagctatcgg	tttgcttccagtaggcttgg	249
CG6554	3R	Female	acctttcctacctttccc	gaatagctaacccagtcatccg	321
CG7840	2L	Female	caagctttccttgcactcgc	tggcccgtcaggttgtcg	739
CG9135	2L	Female	tgcagatcgttgactttgcc	gcagtattgtagttggcagcgg	699
CG5363	2L	Female	gctgcatttagtggcttggg	ctggaaactggaaagtctggc	292
CG3831	2R	Female	caccaagagcacacatagc	ccacgacacaaagtgttctcc	475
CG12276	3R	Female	gcttggttgatgctttcggg	ctggtttaagcggtcttggtgc	496
CG17950	2R	Female	atctctctttgcagctcg	agtgtgagtgggcgacttgg	524
CG5499	3R	Female	gaactttctccaggtgctcg	ctttcatattgaacctcgcc	538
CG10206	2L	Female	cgtacaactaatgccagcc	cgatgacgataaaccttggc	412
CG4299	3R	Female	accgcagtctctgctatcg	cgaaaccaagatggactacc	484
CG4236	3R	Female	agcagcggaatcctttgacg	cgcaatcgtaaatctggc	512
CG5272	3L	Female	gtgtggacgatgatgtaacg	agggtcagttggatgagg	576
CG5757	2R	Female	gagtctgtcaggtacagctc	ctattaagcgtggagcgc	472
CG12314	2L	Female	tgtcacatactcgtcgtcgg	ccatatatacgttcagctcg	355
CG4570	3R	Female	ctcagcatggagtcgaagcg	tgcacaatcgttagctgggc	405
CG12909	2R	Female	gtcacacttaactccacg	cttggtgagctgtgaaggc	746
CG6874	3L	Female	caggtgtactgcggattgg	ctcttacgcttccttctggc	463
CG13690	2L	Female	aaagcggcactcttcgcg	aacaccatcagcaccagc	572
CG3509	3R	Female	cccaatagccctccaagaaagc	ctttacgcatgcgctttccc	371
CG17361	3L	Female	acggtcacactaatcggagg	cgtcgtctaacacgtgtcgg	640
CG32409	3L	Female	aacagacgcgaagcggaagg	agtacctacatcgccacaccg	292
CG6459	2R	Female	gattccatttcgtcgccg	tagaagtctcgcggcgttgc	367
CG3975	2L	Female	gcttgtcgactccgttcactcc	atagctgagcttgcagctcggc	480
CG14434	X	Female	tcctccttgatgccaaacgcc	ttgtttaccatgtgcgaccc	526
CG15717	X	Female	ccttgacacttatcaccc	agcacctggcacctagttcg	702
CG9125	X	Female	aatcgccaccaagaacaaac	taccagtcgggaaggatctg	335
CG3024	X	Female	cgtaactgatcgagttcgcg	agttcgcaaagcagatcccg	660
CG8326	X	Female	gagtggattgcgcatgttgg	cacgagttcgcattgggaggc	146
CG3704	X	Female	aaccagaaagggcaggcg	caactggaggacatcaagc	560
CG12117	X	Female	cgcgaacatgaatctgagcc	ctcgaggacttcgatgaagc	738
CG3004	X	Female	tgccgccaaatagcgtccg	gaatgcgttggacagaacacc	426
CG4593	X	Female	cacgagcaacgtagtccagc	gcatcatagttggtacgc	587
CG8675	X	Female	cgaattccgagtcatcctcg	tccacaatgagcagccagc	645
CG11130	X	Female	tgtgtggactccagctacc	gatgttcgtgtatccgatcccc	317
CG2222	X	Female	gcgcagtgtctcaaagatga	cctgcccctctggtacataa	220
CG9915	X	Female	atagtgccattttgcgaccc	gacctaagtcaacagtcg	228
CG1749	X	Female	gtagatctcgtcctaagc	atccgccaccaactcaatcc	521
CG7931	3R	Male	ggttaggatttctgagggc	tgtagaatactcctcgtcc	391
CG7929	3R	Male	catagtacccaccgtgaagc	ttctaccatgtggtgcctcg	445
CG10252	3R	Male	caaatacgcgccaggtcc	ggtagagcctcattctatgc	215
CG14926	2L	Male	cagaacgaaagggaatcgaa	atcctcaagcgggatacctt	232
CG17956	3R	Male	cgaatcaatcatgtgttgcg	tctaacaacggaagcagc	217
CG17376	2L	Male	cggatctcaacttacatgg	ccaactctcggaccaatcg	279
CG1980	3R	Male	cacgactctggaggtagtac	cgcctgttcttcacatttcc	393
CG5565	2L	Male	attgcgatcgagaccttggc	ctctgtcatggatctcagc	569

CG18418	3L	Male	tgcaactgacctcgtactcg	gcggaagagcttgttgtagg	341
CG6980	3R	Male	tgcaggactcgaatctgcgg	cgctaatatccgctcttcatgc	297
CG6255	3R	Male	tggccatcagatagtcggc	ggacaccaaggtcatggtc	655
CG6332	3R	Male	gcgaacgattgaagaagctc	gcgaagagggcttgtacttg	321
CG3483	2R	Male	ggtcaccatgatcatgggc	cgcatcacatcgccgtaca	656
CG10307	2R	Male	gcagcgccagactaatcta	ccacagatttatgcgttctac	335
CG10750	2L	Male	gtcgaggtgaacggattcat	gtcggagaccttcaccacat	222
CG3085	2R	Male	tactctgcaggatatccg	attctccgccaggagcttcacg	550
CG8564	3L	Male	accttagtgcgagagtactc	gccctcgagatcaactggat	319
CG11475	2R	Male	ggataacggccttgctgcc	cgaagcttcatcctgtgcg	333
CG7387	3L	Male	ggtcgaactcgttgctcttc	acgcctacaacatcctcacc	249
CG18266	2L	Male	acaaagcaggaggaggagcg	ccggtcttgggaggatcaatg	184
CG9314	2L	Male	gcaggtgatgctcaacgagg	acttggagatcctggccg	679
CG6971	3R	Male	taaagctcacgccgcacagg	aggacataacaggatcgc	374
CG7251	2L	Male	agggtggatcgataggac	caccgtgagcctgatgctgcct	261
CG9531	2L	Male	gtgtcctcaataccagcg	attggaacgggcagctggtcg	222
CG6130	3R	Male	ctaataaggaaaccctggc	cacctgctcaatctttgcc	397
CG5045	2L	Male	cgcaacatcaacctgattcc	aaatccaacctgtgcgccgcc	401
CG5276	3R	Male	caggtacaacgaaaccaagg	gttgccacgggctttgtacg	568
CG15179	3R	Male	cgttggcttggccattga	agtggcagtcgcagtttgag	531
CG8277	3L	Male	cagccgtttggactgctaacg	gtacactctctgtattccg	191
CG14717	3R	Male	ccatcgtggtgatgcacg	cgatgtttgagctggtcacg	650
CG11037	3L	Male	cgtcgatgtgggaaagctgg	caacatccgtgtagacgcc	749
CG6036	3R	Male	gaaatacacatgggtggc	gtgataccagctgctcgcagc	621
CG13527	2R	Male	cgagttctgtcggtcacgg	cgtgtagacagatggcag	549
CG11379	X	Male	tcgccttacagctctgggcc	ttcgtctcggagttaggcgg	602
CG15208	X	Male	cgagatggaaagagagcgcg	gacgactgtccatcagacgg	488
CG15035	X	Male	tcgctcagcacctcgagcagc	caatctatactccgctcaccc	533
CG12684	X	Male	gccatgtccatgtcatgtcc	agcacaacacccagagttcc	597
CG2574	X	Male	ctggacggctggcgttaatgg	ctgttgctcatgagtctcgg	405
CG1314	X	Male	caagaaggagatcgacgagc	tcttgcccagacccatctcg	553
CG3708	X	Male	ctatttgagttcggagcagg	gctggatctggagagtaacg	562
CG9156	X	Male	gagaagagcgtaaccagc	ctgagggcgaaattcgaggc	711
CG12681	X	Male	catcgttcacgatcatcccg	ggctagttattgagtcagcc	535
CG12395	X	Male	cagcgtcacagcagaatcgc	ctgaagagctgcccgacgtgc	244
CG13759	X	Male	cggagtggtgcttaatggc	ccgcatccgtatacttgtcg	367
CG2577	X	Male	ggtaatggatctattggg	gtggaacttcatcatcagc	654
CG1950	X	Male	ctcgaagatgggcatcagc	cgtctgtacgagctggacgg	395
CG1503	X	Male	gggtctataaatcagggagg	gttgctgcagtcctcgagcg	278
CG6789	X	Male	gaacagagaatcaagcacgc	tgagcaacacatcgatggcc	727
CG11697	X	Male	cttctttcgttggtagtgggc	ttagccctagagtgctgtccc	576
CG5662	X	Male	ctatctgctgcagtgcgg	agcagaggctcacctcgtgc	448
CG6999	X	Male	aggcgctgaggaagaaagcg	ccttgcgagcctgtgacacttc	556
CG5334	X	Male	gtgagcattgcccataccgc	ctctgtagctttcgagtacgc	630
CG18341	X	Male	cttaccctccaccttgcagc	ccacgcagaggtcgatgttcg	425
CG10920	X	Male	ctgtgccctgtgttataccg	ggatcatgatcacgtggaagg	578
CG7860	X	Male	gcaatccccttaagacgg	ggatctactggatgaatggc	467
CG11981	3R	Unbiased	catcatctagtccatgcgg	aacggaggatgtgttgtggc	605
CG5919	3R	Unbiased	acttccgatacctcacccg	gcatattgagtatcgtgcgc	558
CG5915	3R	Unbiased	agtacatcgaaatggccagc	tgctccagggcattcttagc	603
CG9893	2R	Unbiased	cagcagtctgttaggcagg	ccagactgacatcattccc	673
CG13189	2R	Unbiased	agctctgtggtatggacagc	cagatgccaccacatcagc	404
CG10853	3L	Unbiased	tcatccgcctggatctgcg	tctccctgtgtgtgagccc	291
CG6913	3R	Unbiased	gcaagttgataaggactcgg	ccaaacagaacagacccaag	258
CG9437	2R	Unbiased	cgtcagtccagagttgctcg	gtcaatgccgacatcatcaacg	333
CG8392	2R	Unbiased	cagaaccgagtaactcttaccc	tggccgtcgaatttgatgg	483

CG7953	2L	Unbiased	gtgctatctctagttgcc	gatcacagtccacgacgatcgg	738
CG13419	3R	Unbiased	ccgtgatacatgtcctgc	ggagttatgtgactgtgacgg	435
CG7484	3L	Unbiased	attttggaggtctgcacctg	gtcggcatcctctaccacac	270
CG9283	3L	Unbiased	agtgttcagtcagagtcgcc	acggacatggacatggcgtggg	540
CG13934	3L	Unbiased	gtggtttgtaggcacttgcc	cggtcagcatggcatatgg	518
CG17404	3R	Unbiased	cagtaaaggttgacactcgg	tgcagtcgaattacgtgggc	411
CG10623	2L	Unbiased	gtaacagcctctgcctctagc	gtgagcaacagattcaggagc	541
CG3652	2L	Unbiased	tggaagaatgagctgaaggc	gggattgtacgtcatcac	318
CG9617	3R	Unbiased	tacatctgtgtctccggtcg	gagaaagacaaggcggtggc	323
CG6981	3L	Unbiased	gtttccaccactcagttccc	ctctactgcgtgtagtcgg	292
CG9822	2R	Unbiased	ctgctgtcaatcacgctatg	gatggtacagttcatgtggc	459
CG13845	3R	Unbiased	aacccctaccgatcctcacc	ctagtgcgtctcgccttcg	706
CG3683	2R	Unbiased	actcatttcagctgccgc	agctactgtggatgctgcc	453
CG3476	2L	Unbiased	gtgacctacgaattcctacagg	gctccacaccaaagaatacagc	366
CG6094	2L	Unbiased	ggcagcgacaataagttcaacg	agaagtccactactccactg	447
CG8844	2L	Unbiased	catcaagtgtatgtgccg	catcagctcaaacgaccg	244
CG7508	3R	Unbiased	gatttgatgctgttcggc	actgggctcctagttacagg	419
CG16985	3L	Unbiased	gctccagagcactgtaatcg	gtcgatagtcaagcaacg	370
CG11785	3R	Unbiased	tgaaagggagaatgctgg	aacgcgtccaataccaac	356
CG10035	3R	Unbiased	ccacttagtttacgagcc	gtcccattgtcgtccagacc	492
CG18553	3R	Unbiased	caagacccatggtgttgtgc	gatgaacagcatgacgaaggc	520
CG14629	X	Unbiased	gcaaggatgtagatgcctcagc	gacgaagaccatcaagcttcc	715
CG1885	X	Unbiased	cgtagctatcgctctctcc	cgaggagaacaagatgagc	564
CG1751	X	Unbiased	aacaagtgggacggatcgg	tccaggacgatgccgttctg	463
CG15247	X	Unbiased	tgggcgttcttctagcgtgc	acttccacgctcaactcg	605
CG14227	X	Unbiased	cttggatgctatttcgcag	gagaacctttatgtccgcc	399
CG9919	X	Unbiased	cgatctcataagaaccgg	gtccttagtagtctttggc	501
CG9538	X	Unbiased	tcaagtggaacgacgaactg	tggtagcagcgtaattgcag	372
CG2555	X	Unbiased	ctccatcagtcgagatccg	gtagttgccattggcatcgc	381
CG1397	X	Unbiased	ggactaatctaggatcgg	tgcgtcgctgctaccaatgc	390
CG9571	X	Unbiased	cagaacaacgacaagctcgacg	ggcaggattcatgcgctgg	608
CG14797	X	Unbiased	ctcttccgatcaccatccg	cgttggtggaggcggtgcta	199
CG3603	X	Unbiased	gattgccgctgatcgtaacc	cggtgacatcgatggcagc	631
CG14772	X	Unbiased	cggcaatgggaatggaagc	ctactgtgctttctcctg	376
CG11126	X	Unbiased	ctggcaggacgaactggac	ctggaggaatgggcactcg	285
CG15313	X	Unbiased	actttccacatccgctgaac	atcagtctagtcccggcaga	170
CG9723	X	Unbiased	accaaaccacgtagtccc	gcgaaagaagattccactgtcg	373
CG9164	X	Unbiased	gattactgtcaggtgaggc	atgacgatacgctggtgtcg	546
CG15336	X	Unbiased	cgtgcgtctaacaatagtgc	tgtcgcccgctgcctcttctgc	500

[a]Chromosomal location in *D. melanogaster*.
[b]Sex-biased expression category in *D. melanogaster*.
[c]Length of amplified fragment.
