# Supplementary material for: Molecular evolution of sex-biased genes in the Drosophila ananassae subgroup
Source: BMC Evol Biol. 2009 Dec 16;9:291. doi: 10.1186/1471-2148-9-291 (PMC2809073; doi:10.1186/1471-2148-9-291)
Supplement: Additional file 2 — Sex-biased gene expression in D. ananassae compared to D. melanogaster and D. pseudoobscura. The degree of sex-biased gene expression is given as the ratio of male/female expression. For D. ananassae, gene expression ratio was determined from up to two independent experiments. For D. melanogaster, expression data from up to three independent experiments were used. [file 1471-2148-9-291-S2.pdf]

Additional file 2 – Sex-biased gene expression in *D. ananassae* compared to *D. melanogaster* and *D. pseudoobscura*

Gene	Bias *D. melanogaster*	M/F *D. melanogaster*[a]	Bias *D. ananassae*	M/F *D. ananassae*	Bias *D. pseudoobscura*	M/F *D. pseudoobscura*[e]
**CG10035**	U	1.06	F	0.88[d]	U	1.00
**CG10206**	F	0.18	F	0.37[c]	/	/
**CG10252**	M	35.16	M	15.14[d]	M	8.69
**CG10623**	U	0.95	M	1.29[d]	U	0.97
**CG10750**	M	12.73	U	0.91[c]	M	2.71
**CG10853**	U	0.99	U	1.05[b]	/	/
**CG10920**	M	4.91	M	4.82[d]	M	2.50
**CG11126**	U	1.01	U	1.20[b]	/	/
**CG11130**	F	0.40	U	0.96[b]	F	0.61
**CG11379**	M	3.04	M	1.77[d]	M	1.98
**CG11697**	M	4.81	M	1.94[b]	/	/
**CG11785**	U	1.07	U	0.90[b]	/	/
**CG11981**	U	1.03	F	0.72[d]	U	1.02
**CG12117**	F	0.38	U	0.83[b]	F	0.80
**CG12276**	F	0.27	F	0.60[b]	U	0.99
**CG1239**	F	0.34	U	0.91[b]	F	0.68
**CG12395**	M	7.11	M	1.31[d]	M	2.65
**CG12681**	M	12.14	M	2.53[b]	/	/
**CG12684**	M	6.17	F	0.43[c]	/	/
**CG12909**	F	0.26	U	1.13[d]	F	0.58
**CG1314**	M	8.95	M	1.67[b]	M	1.56
**CG13189**	U	1.03	U	1.18[d]	U	1.01
**CG13419**	U	0.98	U	1.12[b]	U	1.04
**CG13690**	F	0.18	M	1.60[d]	F	0.67
**CG13845**	U	1.02	U	0.98[b]	/	/
**CG13934**	U	0.92	F	0.84[d]	U	1.00
**CG1397**	U	0.97	U	1.04[b]	U	1.00
**CG14227**	U	0.78	M	2.65[d]	/	/
**CG14434**	F	0.24	U	0.88[b]	U	0.96

**CG14629**	U	1.09	M	1.47[d]	U	1.02
**CG14717**	M	16.29	U	1.45[b]	/	/
**CG14797**	U	1.05	U	0.99[b]	U	1.00
**CG14926**	M	25.19	M	3.74[b]	M	4.74
**CG1503**	M	6.16	U	1.24[b]	M	1.49
**CG15035**	M	10.10	U	1.17[d]	/	/
**CG15179**	M	14.09	M	2.13[d]	M	5.90
**CG15208**	M	15.34	M	2.71[b]	M	9.22
**CG15247**	U	1.01	U	1.01[b]	U	1.01
**CG15313**	U	0.99	M	5.21[c]	/	/
**CG15336**	U	1.08	U	0.76[d]	U	1.01
**CG15717**	F	0.28	F	0.69[d]	F	0.62
**CG16985**	U	1.10	F	0.69[b]	U	1.00
**CG17361**	F	0.32	U	0.95[b]	/	/
**CG17376**	M	12.50	M	10.82[d]	/	/
**CG17404**	U	0.93	U	1.07[b]	U	0.99
**CG1749**	F	0.42	U	0.97[d]	U	0.98
**CG1751**	U	1.03	U	1.12[d]	M	1.34
**CG18266**	M	13.68	M	1.94[d]	M	4.81
**CG18341**	M	4.66	U	1.14[b]	M	2.52
**CG18418**	M	16.06	M	2.21[b]	/	/
**CG18553**	U	1.06	U	1.06[b]	U	1.02
**CG1885**	U	0.98	M	2.49[d]	U	0.98
**CG1950**	M	6.68	U	1.24[b]	/	/
**CG2222**	F	0.40	F	0.74[d]	U	0.94
**CG2555**	U	1.00	M	1.94[d]	U	0.99
**CG2574**	M	7.34	M	1.69[b]	U	1.03
**CG2577**	M	8.26	M	1.48[d]	M	1.71
**CG2867**	F	0.30	U	0.75[d]	F	0.67
**CG3004**	F	0.37	U	0.82[b]	F	0.67
**CG3024**	F	0.35	M	3.43[d]	F	0.63
**CG3085**	M	19.81	M	2.20[d]	M	5.20
**CG32409**	F	0.26	U	0.70[b]	F	0.50

**CG3476**	U	0.98	M	24.59[d]	U	0.97
**CG3481**	Control (*Adh*)	-	F	0.69[b]	M	2.09
**CG3483**	M	10.77	M	2.55[b]	F	0.16
**CG3509**	F	0.13	F	0.49[b]	U	0.97
**CG3603**	U	0.98	U	0.68[b]	U	1.02
**CG3652**	U	0.98	U	0.98[b]	F	0.26
**CG3661**	Control (*RpL23*)	0.81	F	0.55[d]	U	0.99
**CG3683**	U	1.04	U	0.74[b]	U	0.97
**CG3704**	F	0.33	U	1.04[b]	M	1.41
**CG3708**	M	5.52	M	1.52[b]	F	0.43
**CG3831**	F	0.32	F	0.65[d]	U	0.96
**CG3975**	F	0.37	U	0.85[b]	U	1.00
**CG4236**	F	0.32	U	0.71[b]	F	0.36
**CG4299**	F	0.24	F	0.46[b]	F	0.66
**CG4570**	F	0.10	U	0.89[b]	F	0.58
**CG4593**	F	0.37	M	4.38[d]	U	0.96
**CG4973**	F	0.32	U	0.95[d]	M	2.77
**CG5045**	M	19.81	M	1.58[d]	F	0.32
**CG5272**	F	0.08	F	0.42[d]	M	1.53
**CG5276**	M	5.67	U	1.19[b]	/	/
**CG5334**	M	3.93	M	1.29[c]	F	0.52
**CG5363**	F	0.21	F	0.58[b]	F	0.36
**CG5499**	F	0.24	F	0.50[d]	/	/
**CG5565**	M	20.83	M	4.39[d]	/	/
**CG5662**	M	4.06	U	1.08[b]	F	0.69
**CG5757**	F	0.26	F	0.63[b]	/	/
**CG5915**	U	1.00	M	1.90[d]	U	1.00
**CG5919**	U	0.98	U	1.21[d]	M	4.58
**CG6036**	M	13.66	M	2.19[d]	F	0.75
**CG6094**	U	1.00	U	0.92[b]	M	2.88
**CG6130**	M	8.22	M	1.95[b]	U	1.06
**CG6255**	M	17.96	U	1.40[d]	M	6.08
**CG6332**	M	23.88	M	4.61[d]	F	0.47

**CG6459**	F	0.27	F	0.52[b]	F	0.38
**CG6554**	F	0.24	U	1.11[b]	U	1.04
**CG6789**	M	7.40	M	1.88[d]	U	1.02
**CG6913**	U	1.00	U	1.16[b]	M	2.11
**CG6971**	M	9.72	M	1.89[b]	M	2.43
**CG6980**	M	8.08	M	2.96[d]	U	0.99
**CG6981**	U	1.03	U	1.06[d]	/	/
**CG7251**	M	11.14	U	1.36[b]	M	1.78
**CG7387**	M	19.63	F/M[f]	0.95[d]	U	1.00
**CG7484**	U	0.95	F	0.63[c]	U	1.00
**CG7508**	U	1.11	U	0.83[d]	U	0.96
**CG7840**	F	0.18	F	0.44[b]	U	1.01
**CG7860**	M	3.11	U	1.08[b]	U	0.97
**CG7953**	U	1.07	U	0.79[b]	M	3.35
**CG8277**	M	19.91	M	4.78[d]	U	0.95
**CG8326**	F	0.34	U	0.82[b]	U	1.02
**CG8392**	U	0.95	U	0.88[b]	M	4.27
**CG8564**	M	35.21	M	2.35[d]	F	0.72
**CG8675**	F	0.42	U	0.93[b]	U	0.98
**CG8844**	U	1.04	M	1.23[d]	F	0.63
**CG9125**	F	0.31	U	0.89[b]	F	0.31
**CG9135**	F	0.16	F	0.35[c]	U	1.02
**CG9164**	U	1.02	U	1.01[b]	F	0.50
**CG9273**	F	0.22	U	0.74[b]	U	1.00
**CG9283**	U	0.98	U	1.12[b]	M	2.99
**CG9314**	M	24.06	M	2.64[b]	/	/
**CG9383**	F	0.28	U	0.98[d]	U	0.99
**CG9437**	U	0.95	U	1.05[b]	M	1.84
**CG9531**	M	3.48	M	1.74[b]	F	0.81
**CG9538**	U	0.82	F	0.86[d]	/	/
**CG9617**	U	0.98	U	1.08[b]	/	/
**CG9723**	U	0.95	U	0.86[b]	U	0.98
**CG9893**	U	0.95	U	0.96[b]	F	0.59

**CG9915**	F	0.43	U	0.83[d]	U	0.99
**CG9919**	U	1.02	U	1.00[b]	U	1.00

[a]Average male/female expression ratio from Parisi *et al.* (2003), Ranz *et al.* (2003), and Gibson *et al.* (2004).
[b]Male/female expression ratio from Zhang *et al.* (2007).
[c]Male/female expression ratio from PCR-amplicon microarrays (this study).
[d]Average value from Zhang *et al.* (2007) and PCR-amplicon microarrays.
[e]Male/female expression ratio from Zhang *et al.* (2007) ("/" indicates genes with either no ortholog in *D. pseudoobscura* or for which expression data are not available).
[f]This gene showed female-biased expression in our microarray experiment, but male-biased expression in the experiment of Zhang *et al.* (2007). The mean of the two experiments is given in the table.
