# Supplementary material for: Molecular evolution of sex-biased genes in the Drosophila ananassae subgroup
Source: BMC Evol Biol. 2009 Dec 16;9:291. doi: 10.1186/1471-2148-9-291 (PMC2809073; doi:10.1186/1471-2148-9-291)
Supplement: Additional file 3 — Comparison D. ananassae/D. melanogaster. For 43 genes, intraspecific polymorphism data were available for both D. melanogaster (Zimbabwe, Africa) and D. ananassae (Bangkok, Thailand). For D. melanogaster, divergence was determined to D. simulans. For D. ananassae, D. atripex and/or D. phaeopleura were used for calculating divergence. [file 1471-2148-9-291-S3.pdf]

Additional file 3 – Comparison *D. ananassae*/*D. melanogaster*

		D. ananassae									***D. melanogaster***							
Gene	Bias[a]	D_S[b]	P_S[c]	D_N[d]	P_N[e]	*P*- value[f]	α[g]	TD_{syn}[h]	TD_{non}[i]	Outgroup	D_S[b]	P_S[c]	D_N[d]	P_N[e]	*P*- value[f]	α[g]	TD_{syn}[h]	TD_{non}[i]
**AUTOSOMAL**																		
CG10035	FU	32	11	25	3	0.112	0.651	0.235	-1.642	Dph	25	5	9	4	0.3100	-1.222	-0.99	0.50
CG10252	MM	28	13	6	3	0.925	-0.077	0.164	-0.804	Dat	19	3	0	0	na	na	-0.11	na
CG11981	FU	19	8	0	0	na	na	0.438	na	Dat	15	1	0	0	na	na	0.33	na
CG12276	FF	43	10	17	7	0.321	-0.771	0.451	0.066	Dat	41	3	8	5	0.0081	-7.542	0.02	-0.91
CG1239	UF	45	6	22	3	0.976	-0.023	-0.447	-1.187	Dat	23	5	18	2	0.4386	0.489	-0.99	-0.03
CG14717	UM	43	28	40	5	0.001	0.808	0.124	-1.014	Dph	23	11	7	6	0.3800	-0.792	-0.18	-1.43
CG3509	FF	59	15	33	3	0.095	0.642	0.131	0.278	Dat	26	13	35	6	0.0480	0.657	0.36	-0.24
CG4973	UF	40	9	5	12	0.004	-5.000	-0.194	0.247	Dat	43	8	9	9	0.0053	-4.375	-0.02	-1.81
CG5499	FF	6	0	0	0	na	na	na	na	Dph	7	3	0	3	0.0699	na	0.20	-1.45
CG5915	MU	13	4	0	2	0.088	na	0.585	-1.461	Dph	9	3	1	0	1.0000	1.000	1.19	na
CG6036	MM	53	9	11	0	0.338	1.000	0.376	na	Dat	26	21	11	5	0.3406	0.437	0.32	-1.70
CG6971	MM	26	3	1	0	1.000	1.000	-1.111	na	Dph	26	7	0	0	na	na	-1.33	na
CG6980	MM	24	10	16	1	0.036	0.850	0.618	1.202	Dat	16	5	13	4	0.9839	0.015	0.43	0.18
CG7508	UU	12	33	3	7	0.832	0.152	-0.679	-1.118	Dat	23	4	9	3	0.4543	-0.917	-0.37	-0.42
CG10853	UU	9	4	3	0	0.529	1.000	0.998	na	Dat	6	7	2	4	0.6000	-0.714	0.00	-0.78
		12	3	6	0	0.526	1.000			Dph								
CG18418	MM	33	9	11	4	0.682	-0.333	0.151	-0.893	Dat	27	33	13	5	0.0397	0.685	-0.01	0.25
		33	9	12	4	0.772	-0.222			Dph								
CG5272	FF	25	4	21	6	0.410	-0.786	-1.387	-0.505	Dph	17	4	14	3	0.9110	0.089	-0.90	-0.42
CG6981	UU	17	8	1	0	1.000	1.000	0.139	na	Dat	13	4	0	0	na	na	0.10	na
CG7387	FM	49	16	16	5	0.940	0.043	-0.737	-0.908	Dat	45	12	18	6	0.7721	-0.250	-0.69	-1.07
CG8277	MM	23	12	8	10	0.138	-1.396	-1.45	-1.223	Dat	14	5	24	3	0.1800	0.650	0.12	-0.38
		17	12	5	10	0.109	-1.833			Dph								
CG9383	UF	35	17	8	1	0.259	0.743	-0.424	0.409	Dph	14	1	2	0	1.0000	1.000	-0.09	na
CG13189	UU	42	36	0	3	0.107	na	0.279	-1.627	Dph	26	22	3	0	0.2494	1.000	-0.22	na
CG3085	MM	44	33	3	1	0.468	0.556	-0.297	-1.122	Dph	25	41	5	1	0.0281	0.878	-0.45	-0.07
CG6459	FF	31	11	5	3	0.724	-0.691	-0.268	-0.51	Dat	21	7	5	3	0.4650	-0.800	0.93	-1.75
		28	14	11	3	0.391	0.455			Dph								
CG10750	UM	28	17	10	1	0.047	0.835	0.518	-0.129	Dat	21	20	10	0	0.0039	1.000	-0.33	na
CG18266	MM	53	23	47	10	0.089	0.510	-1.073	0	Dph	35	11	55	9	0.1893	0.479	-0.83	-0.69
CG3476	MU	31	1	6	4	0.004	-19.667	-0.032	-0.162	Dph	17	16	0	6	0.0267	na	-0.99	-1.56
CG7840	FF	32	25	4	3	0.960	0.040	-0.116	-1.56	Dat	18	17	12	4	0.1057	0.647	-0.04	0.42
		31	25	2	3	0.653	-0.860			Dph								

CG9135	FF	51	27	2	3	0.264	-1.833	0.784	-1.550	Dat	28	19	3	0	0.2788	1.000	-0.78	na
		52	27	3	3	0.444	-0.926			Dph								
**X-LINKED**																		
CG10920	MM	61	18	23	3	0.193	0.558	0.518	-1.536	Dat	43	24	56	11	0.099	0.648	-0.06	-0.27
CG11697	MM	0	19	1	3	0.174	1.000	0.041	1.940	Dat	28	1	17	5	0.0733	-7.235	-1.13	-0.32
CG15336	UU	19	6	11	2	0.529	0.424	-0.032	-1.449	Dat	10	17	10	13	0.6400	0.235	-1.47	-0.68
		21	6	8	2	0.883	0.125			Dph								
CG15717	FF	28	23	20	8	0.146	0.513	-0.816	-0.543	Dat	12	7	16	8	0.8107	0.143	-0.90	0.12
		25	23	9	8	0.951	0.034			Dph								
CG1749	UF	35	7	9	0	0.328	1.000	-0.480	na	Dph	26	5	4	9	0.0007	-10.70	-0.94	-0.62
CG18341	UM	1	9	2	4	0.254	0.778	-1.417	0.925	Dat	26	22	18	2	0.0026	0.869	-0.08	-1.42
CG2222	FF	21	12	4	0	0.282	1.000	0.437	na	Dph	10	11	2	4	0.5310	-0.818	-0.31	-1.79
CG2577	MM	39	22	12	2	0.095	0.705	-0.091	-1.436	Dat	32	26	4	0	0.1320	1.000	-0.98	na
		44	22	12	2	0.135	0.667			Dph								
CG3004	UF	26	6	6	10	0.003	-6.222	-0.307	-0.018	Dat	23	18	7	0	0.0360	1.000	-0.43	na
		26	6	9	11	0.007	-4.296			Dph								
CG3024	MF	54	7	37	7	0.512	-0.459	-1.190	-1.189	Dat	35	5	51	6	0.7637	0.176	-0.68	-0.58
		63	7	44	7	0.529	-0.432			Dph								
CG4593	MF	23	7	2	1	0.709	-0.643	-0.692	0.543	Dat	12	19	3	0	0.0760	1.000	0.48	na
		17	7	4	1	0.668	0.393			Dph								
CG9723	UU	64	10	16	7	0.076	-1.800	-0.588	-1.305	Dph	36	18	61	5	0.0003	0.836	-0.46	-1.01
CG11379	MM	21	8	12	1	0.119	0.781	0.028	-1.133	Dat	17	7	5	4	0.4130	-0.943	-1.07	-1.39
		23	8	7	1	0.401	0.589			Dph								
CG1314	MM	43	24	13	5	0.518	0.311	-0.194	0.286	Dph	33	12	80	4	0.0004	0.863	-1.30	-0.82

[a] First letter indicates expression in *D. ananassae*, second letter expression in *D. melanogaster* (M=male-biased, F=female-biased, U=unbiased).
[b] The total number of synonymous fixed differences.
[c] The total number of nonsynonymous fixed differences.
[d] The total number of synonymous polymorphisms.
[e] The total number of nonsynonymous polymorphisms.
[f] *P*-value of McDonald-Kreitman test. Red indicates a relative excess of nonsynonymous divergence (positive selection), blue indicates a relative excess of nonsynonymous polymorphism.
[g] $\alpha = 1 - [(D_S*P_N)/(D_N*P_S)]$.
[h] Tajima's *D* at synonymous sites.
[i] Tajima's *D* at nonsynonymous sites.
