# Supplementary material for: Molecular evolution of sex-biased genes in the Drosophila ananassae subgroup
Source: BMC Evol Biol. 2009 Dec 16;9:291. doi: 10.1186/1471-2148-9-291 (PMC2809073; doi:10.1186/1471-2148-9-291)
Supplement: Additional file 4 — Inference of ancestral sex-biased expression state of genes differing in expression between D. melanogaster (Dmel) and D. ananassae (Dana), using D. pseudoobscura (Dpse) as the outgroup. For 47 genes showing conflicting sex-biased classifications between D. melanogaster and D. ananassae, we inferred the ancestral expression state using microarray data from D. pseudoobscura. [file 1471-2148-9-291-S4.pdf]

Additional file 4 – Inference of ancestral sex-biased expression state of genes differing in expression between *D. melanogaster* (*Dmel*) and *D. ananassae* (*Dana*), using *D. pseudoobscura* (*Dpse*) as the outgroup

Gene	*Dana*	*Dmel*	*Dpse*
**CG10035**	F	U	U
**CG11981**	F	U	U
**CG13934**	F	U	U
**CG16985**	F	U	U
**CG7484**	F	U	U
**CG9538**	F	U	F
**CG10623**	M	U	U
**CG14629**	M	U	U
**CG1885**	M	U	U
**CG2555**	M	U	U
**CG3476**	M	U	U
**CG8844**	M	U	U
**CG7387**	F/M	M	M
**CG13690**	M	F	F
**CG3024**	M	F	F
**CG4593**	M	F	F
**CG11130**	U	F	F
**CG12117**	U	F	F
**CG1239**	U	F	F
**CG12909**	U	F	F
**CG14434**	U	F	U
**CG17361**	U	F	U
**CG1749**	U	F	U
**CG2867**	U	F	F
**CG3004**	U	F	F
**CG32409**	U	F	F
**CG3704**	U	F	U
**CG3975**	U	F	U
**CG4236**	U	F	U
**CG4570**	U	F	F
**CG4973**	U	F	U
**CG6554**	U	F	F
**CG8326**	U	F	U
**CG8675**	U	F	F
**CG9125**	U	F	F
**CG9273**	U	F	F
**CG9383**	U	F	U
**CG9915**	U	F	F
**CG10750**	U	M	M
**CG1503**	U	M	M
**CG15035**	U	M	U
**CG18341**	U	M	M
**CG5276**	U	M	M
**CG5662**	U	M	U
**CG6255**	U	M	U
**CG7251**	U	M	U
**CG7860**	U	M	U
