# Supplementary material for: Molecular evolution of sex-biased genes in the Drosophila ananassae subgroup
Source: BMC Evol Biol. 2009 Dec 16;9:291. doi: 10.1186/1471-2148-9-291 (PMC2809073; doi:10.1186/1471-2148-9-291)
Supplement: Additional file 5 — PCR and sequencing primers. Primers were designed to amplify genomic regions of 43 protein-coding genes. The PCR primers were also used as sequencing primers, with internal primers designed when necessary. [file 1471-2148-9-291-S5.pdf]

Additional file 5 – PCR and sequencing primers

Gene	Forward primer (5'-3') (F)	Reverse primer (5'-3') (R)	Internal Primer
**CG10035**	ggaaatcttcgacgaagacact	gtgaaatcggttttatggcgt	
**CG10252**	tctaatcaagtaggcctggc	aaccgcaaggcaaagtagc	
**CG10750**	ctatcataataattgacacca	atgcgatcaatctcatggct	
**CG10853**	acacctgtgcgaatcagatg	ttctccctgtgtgtgagcc	
**CG10920**	tgcgagtgtgagacagacatcg	caggctgctccgaattcagtc	
**CG11379**	tttctgtctctgccagaagc	agcggttggcattacttaaccc	
**CG11697**	acactgtcaacagagatgcc	agcaacaaactgccaccttgc	
**CG11981**	aagctgtcagttgccagac	tagtgggttcattggcggtgc	
**CG12276**	gaagcgttagtgctttttggc	gataagttcgggacattaaaaacg	
**CG1239**	tcaatggtcacctgtggttagt	taaacgcgcaaaacagcact	
**CG1314**	tttagtggtgtggctctaatcagt	ccgatccgtaaaaacctgagaa	ctcatcttcgacagtccg (F)<br>ttctgctcctgctcctgc (R)
**CG13189**	agagctcctcagttgaaagc	acagatgccaccacatcagc	
**CG14717**	aaagccatgttttcttaccctt	gctgaaatttcaggaaactccc	
**CG15336**	agatttggcacacgcagtgc	acaccgtttggtcccactc	
**CG15717**	cagctatcgccgatcttcgc	aaggacctaccccaatcaccc	
**CG1749**	cgacggcagagaaattgaaa	cgaaaatagagcattcatttacag	
**CG18266**	tctcaaacacatcaggtcgc	actacctaggcacttcaaccg	
**CG18341**	acacattcaaaaccttctgc	tgtgcggtaaattagcatgc	
**CG18418**	acggttaacatagggactttgct	cattttgcatatacatccgg	
**CG2222**	tctataaccggtatttgaaccgg	tgatggacatggattgtt	
**CG2577**	ttagttccttgcgcccggttcg	gaatcgagaggccttctggg	
**CG3004**	tgaatgcagctggtcacact	ggttaccctataaagaccataacg	
**CG3024**	tcgcgattaggtcacactagc	taaccaaggatctgggaccg	ctacgtatccaagtacctcgg (F)
**CG3085**	cagttgggaggacaaattcagt	cgagggggattacatactttta	
**CG3476**	aggttggcaggtcttaaaccg	gcataattctggttgattcatgg	
**CG3509**	atcatccgccggtgcagtagtg	tcagaccaagcaactgccctggc	agaggcggcgaaggataagg (F)<br>tagtgacctttgccctctc (R)
**CG4593**	tcgataactgtcagctggaaagc	aattgggaggacagctgagagg	
**CG4973**	ttaccacctctagcagtcgg	tttaggtagtaactgttgg	
**CG5272**	atgcctggagccactatatg	tcagatcgctctactttaacc	cacaatagaaccagtggcagca (F)<br>agggctcatagtggactcct (R)
**CG5499**	aatggctccttctctgacgg	tgcgacagaatgacgttgcc	
**CG5915**	agtccagtcgtgttatctgc	agaggccattaaggagtctgc	
**CG6036**	tctagatctaattggcttcgg<br>gaggatcgacggcaatatgttg	ataacgcacagctgctgcc<br>aacccgctttatcatcacactgc	
**CG6459**	taagcgacaaccctagttgc	ttgaagctcctcctattccg	
**CG6971**	cagagccagaaactcatttgtgt	gccaagatggaggagcttaag	atctccaggagatgctggacac (F)<br>gacatggcgatctcatcgcggat (R)
**CG6980**	cttcggttgctatagcatcc	acagatttgggcagtgttcacc	
**CG6981**	tgatatagtgctatccagtg	ttgtttattcggcgattgc	
**CG7387**	caatcggacaagttgtaggc	gaacaacaagatcctccagc	
**CG7508**	tgcctgattgcctgccatcg	ctactgggctcctagttacagg	
**CG7840**	tcgacattcaccgcgacaagc	atcgactgcagcctgatcc	
**CG8277**	tgtaagcaattcccactcgg	cgaagccaatgtcatcttcc	
**CG9135**	agccgcttgtgcgttacgctttgc	agcagcagatagccactttcggg	tggttcggtaacccacttggagc (R)
**CG9383**	acaaggcggagccataaacag	agggtacgaggaggactatg	
**CG9723**	tatgttccaaacaaaccgctg	tgcgtatttggcatttctgc	
