# Supplementary material for: Molecular evolution of sex-biased genes in the Drosophila ananassae subgroup
Source: BMC Evol Biol. 2009 Dec 16;9:291. doi: 10.1186/1471-2148-9-291 (PMC2809073; doi:10.1186/1471-2148-9-291)
Supplement: Additional file 6 — All additional material. All files can be accessed through index.html, which can be viewed with any standard internet browser. [file 1471-2148-9-291-S6.zip › index.html]

Additional File


# Molecular evolution of sex-biased genes in the *Drosophila ananassae* subgroup

## Sonja Grath, John F Baines, John Parsch

### Department of Biology, University of Munich (LMU), Munich, Germany

# Additional Material

## Microarrays

### Gene selection and PCR primers

Our  *D. ananassae*  microarrays contained PCR-amplified exon probes to 148 genes, with each probe spotted in 8 replicates. 136 of these genes are comprised of 91 autosomal genes reported by Pröschel *et al*. (2006) and 45 X-linked genes reported by Baines *et al.* (2008). For these genes, polymorphism data from 12 African *D. melanogaster* strains and divergence data to *D. simulans* are available. We used the *D. ananassae* (Assembly August 2005; http://genome.ucsc.edu/)
genome to design polymerase chain reaction (PCR) primers flanking single exons of all of the above genes.

**Additional File 1 - Primers for PCR-amplicon microarrays**  
Download

### Sex-biased gene expression

**Additional File 2 - Sex-biased gene expression in *D. ananassae* compared to *D. melanogaster* and *D. pseudoobscura***  
Download

### Raw data

Raw data for all microarrays can be downloaded below. For the strain KK1 (Kota Kinabalu, Borneo), we performed four biological replicates. For the strain KK2, we performed two biological replicates. Each biological replicate included a technical (dye-swap) replicate, leading to a total of 12 microarrays.

|  |  |  |
| --- | --- | --- |
| **Array** | **Description** | **Data** |
| KK1\_1 | *D. ananassae* strain KK1, biological replicate 1 | Download |
| KK1\_1\_ds | *D. ananassae* strain KK1, biological replicate 1, dye-swap | Download |
| KK1\_2 | *D. ananassae* strain KK1, biological replicate 2 | Download |
| KK1\_2\_ds | *D. ananassae* strain KK1, biological replicate 2, dye-swap | Download |
| KK1\_3 | *D. ananassae* strain KK1, biological replicate 3 | Download |
| KK1\_3\_ds | *D. ananassae* strain KK1, biological replicate 3, dye-swap | Download |
 KK1\_4 | *D. ananassae* strain KK1, biological replicate 4 | Download || KK1\_4\_ds | *D. ananassae* strain KK1, biological replicate 4, dye-swap | Download |
| KK2\_1 | *D. ananassae* strain KK2, biological replicate 1 | Download |
| KK2\_1\_ds | *D. ananassae* strain KK2, biological replicate 1, dye-swap | Download |
| KK2\_2 | *D. ananassae* strain KK2, biological replicate 2 | Download |
| KK2\_2\_ds | *D. ananassae* strain KK2, biological replicate 2, dye-swap | Download |

## Analysis of polymorphism and divergence

### Comparison *D. ananassae*/*D. melanogaster*

For 43 genes, we have polymorphism data for both *D. melanogaster* (Zimbabwe, Africa) and *D. ananassae* (Bangkok, Thailand). For *D. melanogaster*, divergence was determined to *D. simulans*. For *D. ananassae*, divergence was determined to *D. atripex* and/or *D. phaeopleura*.

**Additional File 3 - Comparison *D. ananassae*/*D. melanogaster***  
Download

### Ancestral state of sex-biased gene expression

For genes that differed in their sex-bias classification between *D. melanogaster* and *D. ananassae*, we inferred the ancestral expression state using *D. pseudoobscura* as an outgroup and published microarray data from Zhang *et al.* (2007).

**Additional File 4 - Inference of ancestral sex-biased expression state of genes differing in expression between *D. melanogaster* (*Dmel*) and *D. ananassae* (*Dana*), using *D. pseudoobscura* (*Dpse*) as the outgroup**  
Download

### PCR and sequencing primers

We designed PCR primers flanking the coding regions of each target gene. PCR primers were used for both PCR and sequencing. For some genes, additional internal (Int) primers were used for sequencing.

**Additional File 5 - PCR and sequencing primers**  
Download

### References

Baines JF, Sawyer SA, Hartl DL, Parsch J: **Effects of X-linkage and sex-biased gene expression on the rate of adaptive protein evolution in *Drosophila***. *Mol Biol Evol*. 2008, **25**:1639-1650.  
Gibson G, Riley-Berger R, Harshman L, Kopp A, Vacha S, Nuzhdin S, Wayne M: **Extensive sex-specific non-additivity of gene expression in Drosophila melanogaster.** *Genetics* 2004, **167**:1791-1799.  
Parisi M, Nuttall R, Naiman D, Bouffard G, Malley J, Andrews J, Eastman S, Oliver B: **Paucity of genes on the *Drosophila* X chromosome showing male-biased expression.** *Science* 2003, **299**:697-700.  
Pröschel M, Zhang Z, Parsch J: **Widespread adaptive evolution of *Drosophila* genes with sex-biased expression**. *Genetics* 2006, **174**:893-900.  
Ranz JM, Castillo-Davis CI, Meiklejohn CD, Hartl DL: **Sex-dependent gene expression and evolution of the *Drosophila* transcriptome.** *Science* 2003, **300**:1742-1745.  
Zhang Y, Sturgill D, Parisi M, Kumar S, Oliver B: **Constraint and turnover in sex-biased gene expression in the genus *Drosophila***. *Nature* 2007, **450**:233-238.
